# Syndrome de Perthes: à propos de deux cas pédiatriques

**DOI:** 10.11604/pamj.2020.35.51.16483

**Published:** 2020-02-21

**Authors:** Soufiane El Youssfi, Abderrazak Ou-Meskour, Bachir Belkheiri, Mohamed Abderrahmane Jdoud, Said Benlamkaddem, Mohamed Adnane Berdai, Mustapha Harandou

**Affiliations:** 1 Service de Réanimation Mère-Enfant, CHU Hassan II, Fès, Maroc

**Keywords:** Syndrome de Perthes, compression thoracique, pédiatrie, Perthes syndrome, chest compression, pediatrics

## Abstract

Le syndrome de Perthes est défini par l’association d’hémorragies sous conjonctivales, de cyanose cervico-faciale et des pétéchies à des manifestations neurologiques. La compression brutale du thorax ou thoraco-abdominale bloquée en inspiration forcée avec effort à glotte fermée est le mécanisme responsable du syndrome. Le pronostic est en général bon si la durée de la compression est brève et la réanimation cardiorespiratoire commencée précocement. L’objectif de cet article est de décrire les caractéristiques du syndrome de Perthes chez les enfants.

## Abstract (en)

Perthes syndrome is defined by the association among subconjunctival hemorrhage, cervicofacial cyanosis and petechiae up to neurological manifestations. Violent compression of the thorax or thoracoabdominal block after forced inspiration with glottis closure due to effort is the mechanism responsible for the syndrome. Prognosis is generally good if the compression is brief and cardiopulmonary resuscitation has been started early. The purpose of this study was to describe the features of Perthes syndrome among children.

## Introduction

Le syndrome de Perthes ou syndrome cave supérieur post traumatique, ou asphyxie traumatique, est une pathologie rare secondaire à une compression thoracique sévère.Ce syndrome est caractérisé cliniquement par une cyanose, un œdème, des pétéchies cervico-faciales et une hémorragie sous conjonctivale [1]. Le mécanisme physiopathologique consiste en une hyperpression veineuse cave supérieure survenant au cours d’une compression thoracique ou thoraco-pulmonaire post-traumatique brutale et brève [2]. Les symptômes neurologiques observés, au cours du syndrome, sont dus à la chute brutale du débit sanguin cérébral et de l’asphyxie secondaire à cette compression thoracique [2].

## Patient et observation

**Figure 1:**
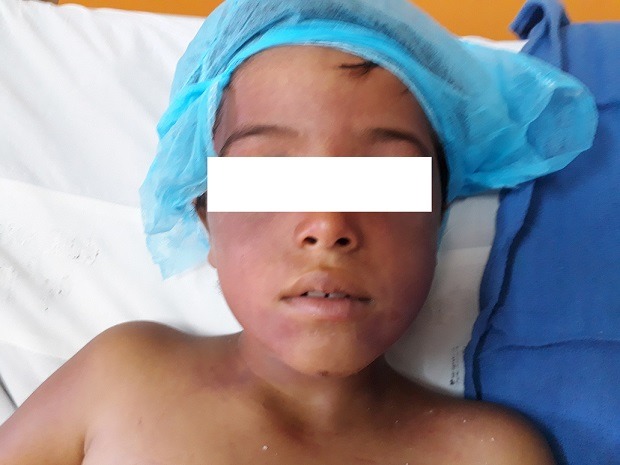
Cyanose cervico-faciale associée à des pétéchies cervicales

**Observation n°1:** un jeune enfant de 8 ans, sans antécédents pathologiques, était admis aux urgences pour prise en charge d’un trouble de conscience à la suite d’un accident de la voie publique (AVP). L’examen initial trouvait un patient inconscient avec un score de Glasgow (GCS) à 11, sans signes de localisation avec des pupilles égales et réactives. Sa tension artérielle était à 117/55 mmHg, et sa fréquence cardiaque était à 120 b/min. L’examen pleuropulmonaire trouvait un patient polypnéique avec une fréquence respiratoire à 30 c/min et une saturation en O2 à 100% sous oxygénothérapie. L’examen abdomino-pelvien trouvait une défense au niveau de l’hypochondre droit, des multiples ecchymoses punctiformes, présence de deux plaies inguinales et une autre au niveau des organes génitaux externes. Par ailleurs, on notait la présence d’une cyanose cervico-faciale, des pétéchies cervicales (Figure 1), d’une hémorragie sous conjonctivale bilatérale et des multiples écorchures au niveau des deux membres inférieurs. La TDM thoraco-abdominale objectivait des foyers de contusion pulmonaire bilatérale, des fractures et contusions hépatiques sans atteinte vasculaire, un hémopéritoine de moyenne abondance et une fracture de la branche ischio-pubienne droite, l’examen ophtalmologique par fond d’œil était normal. Le bilan biologique réalisé aux urgences montrait une anémie normochrome normocytaire à 8,4 g/dl, une élévation du bilan des transaminases hépatiques avec des GOT et GPT à 47 et à 24 fois la normale, respectivement. Le reste du bilan était normal. Le patient était mis sous traitement non spécifique comprenant l’oxygène, le ratio de base, les antalgiques et protection gastrique. L’évolution sur le plan clinique marquait par la disparition de la cyanose en quelques heures, une amélioration progressive de son état de conscience, et les pétéchies régressaient au bout de 2 jours avec une diminution de l’hémorragie sous conjonctivale et de la sensibilité abdominale. Sur le plan radiologique, une échographie de contrôle faite montrait une diminution de l’abondance de l’hémopéritoine. L’évolution sur le plan biologique marquait par l’aggravation de son anémie qui est passé à 6,1 g/dl nécessitant une transfusion par un culot globulaire et une amélioration du bilan des transaminases hépatiques. Le patient a été déclaré sortant à J4 de son admission.

**Figure 2:**
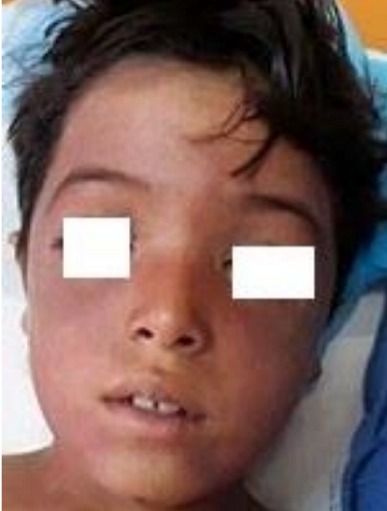
Pétéchies cervico-faciales

**Figure 3:**
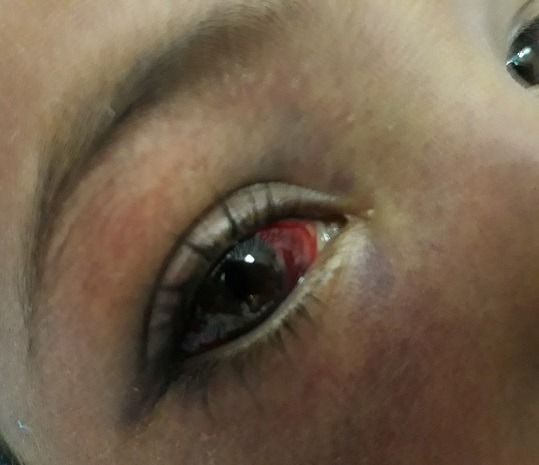
Hémorragie sous conjonctivale

**Observation n°2:** un jeune enfant de 9 ans sans antécédents pathologiques, était admis aux urgences pour prise en charge d’un polytraumatisme à la suite d’un AVP. L’examen initial trouvait un patient inconscient, GCS à 9 sans signes de localisation, des pupilles égales et réactives, hypotendu à 75/50 mmHg, tachycarde à 180 bpm, des extrémités froides avec un temps de recoloration supérieur à 3 secondes. L’examen pleuropulmonaire trouvait un enfant polypnéique, sa fréquence respiratoire était à 34 c/min et une saturation en O_2_ à 60% sous oxygène d’où la décision de son intubation. L’examen abdomino-pelvien trouvait une sensibilité abdominale diffuse avec des multiples ecchymoses punctiformes. Par ailleurs, on notait la présence d’une cyanose cervico-faciale, des pétéchies cervicales (Figure 2) et d’une hémorragie sous conjonctivale (Figure 3). Un scanner thoraco-abdominal montrait un épanchement pleurale bilatéral de moyen abondance plus marqué à gauche, un épanchement intrapéritonéal de grande abondance avec une fracture dévascularisation intéressant la quasi-totalité du foie droit, l’examen ophtalmologique était normal. Le bilan biologique était réalisé aux urgences montrait une anémie normochrome normocytaire à 9,6 g/dl, le reste du bilan était normal. Le patient était mis sous ventilation protectrice, noradrénaline à faible dose, protection gastrique et ratio de base. L’évolution sur le plan clinique marquait par la disparition de la cyanose, des pétéchies et diminution de l’hémorragie sous conjonctivale au premier jour, avec une amélioration de son état de conscience, extubé à J2 avec une bonne adaptation. Sur le plan biologique, l’évolution marquait par une déglobulisation, il était transfusé par deux culots globulaires, l’hémoglobine de contrôle était à 11,3 par rapport à 6.9 g/dl. Une radio du thorax et une échographie abdominale de contrôle montraient une amélioration favorable de son épanchement pleural et abdominal. Le patient a été déclaré sortant à J6 de son admission.

## Discussion

En 1837, Ollivier a décrit pour la première fois l’asphyxie traumatique. Puis Perthes en 1900 a donné une description complète de ce syndrome [3]. Leur incidence est faible (1/20000) avec une prédominance masculine [4]. Les AVP sont la cause la plus fréquente de l’asphyxie traumatique dans la population pédiatrique, les autres causes sont rares comme les accidents de sport, les écrasements, les convulsions épileptiques et l’asthme [4, 5]. Un phénomène de compression ou d’écrasement thoracique est toujours retrouvé dans le mécanisme physiopathologique du syndrome. La compression brutale du thorax ou thoraco-abdominale bloquée en inspiration forcée avec effort à glotte fermée, à cause de réaction de peur, entraine une augmentation majeure des pressions de la veine cave supérieure, une stase veineuse et des ruptures capillaires et veinulaires dans les territoires à faible capacitance [5]. On peut distinguer deux types de lésions: lésions directes en rapport avec le traumatisme lui-même et des lésions indirectes dues à la compression et à l’hyperpression thoracique.L’absence de ces phénomènes dans les territoires sous-jacents serait expliquée par le collapsus de la veine cave inférieure au moment de la manœuvre de Valsalva, protégeant le territoire veineux sous-jacent [6]. La grande élasticité thoracique chez l’enfant permet de prévenir des lésions osseuses costales même en présence des lésions intrathoraciques, ce qui est observé dans nos cas. La durée et le poids de compression influencent dans la survenue des lésions [7]. Les conséquences de ces phénomènes est l’apparition des signes cutanéo-muqueux (SCM),neurologiques, visuels, respiratoires et hémodynamiques. La cyanose, les pétéchies cervico-thoraciques et l’hémorragie sous conjonctivale sont les SCM retrouvés chez plus de 92% des cas décrits [6, 8], tous ces signes sont retrouvés chez nos patients. L’atteinte neurologique, qui fait la gravité de ce syndrome, est fréquente (90%), se traduit par des troubles de conscience jusqu’au coma profond, ces manifestations neurologiques observées sont dues à des phénomènes d’anoxie cérébrale, secondaire à la chute brutale du débit sanguin cérébral et de l’asphyxie dues à la compression thoracique traumatique [9]. Habituellement, ces signes neurologiques régressent au bout d’un à deux jours [10], c’est le cas des deux observations rapportées ou l’atteinte neurologique est améliorée au bout d’un à deux jours. L’asphyxie prolongée, liée à l’absence de la levée rapide d’obstacle et l’absence de la réanimation cardiorespiratoire adéquate, aboutit à une anoxie cérébrale sévère avec des séquelles cérébrales irréversibles [11]. Les signes visuels observés au cours du syndrome de Perthes sont multiples et liés à l’intensité de la compression thoracique traumatique, ces troubles sont plus souvent transitoires et régressent complétement mais lentement [10, 11], nos deux patients ont bénéficié d’un fond d’œil qui était normal chez les deux. Par ailleurs, les lésions thoraco-abdominales non spécifiques qui ont été décrites, témoignent de l’intensité du traumatisme, il peut s’agir d’une contusion pulmonaire, des fractures des côtes, d’une hémopneumothorax, d’une atteinte des articulations, d’une hémoptysie ou d’une rupture diaphragmatique [12], chez nos patients, un a présenté une contusion pulmonaire simple et l’autre présente un épanchement pleural bilatéral. Les lésions cardiaques, sont exceptionnelles; elles peuvent se manifester par une instabilité hémodynamique, à cause du choc cardiogénique secondaire à une contusion myocardique ou de choc hémorragique secondaire à une lésion des gros vaisseaux [13]. Les lésions abdominales peuvent être: un hémopéritoine, une hémorragie digestive, une lacération ou perforation des organes creux [14], les deux cas rapportés ont présenté un hémopéritoine et des lésions hépatiques. Autres lésions peuvent être retrouvées, les lésions génito-urinaires, hypoacousie secondaire à la perforation du tympan, hématurie et des lésions médullaires [15]. La prise en charge doit être commencée au lieu de l’accident par la levée précoce et rapide de compression et la réanimation cardiorespiratoire, et aussi au moment de transport en milieu hospitalier [11, 12]. Le pronostic est en général bon avec une survie de plus de 90% [15], la mortalité était liée à deux facteurs: durée de la compression et la présence des lésions associées [14].

## Conclusion

Le syndrome de Perthes ou asphyxie traumatique est relativement rare. Il est caractérisé par la coloration violacée frappante au niveau de la tête, du cou et de la partie supérieure du thorax avec une hémorragie sous-conjonctivale massive. Dans la majorité des cas, il résulte de la compression thoracique ou thoraco-abdominale. Le diagnostic de ce syndrome repose essentiellement sur la constatation du “masque ecchymotique”, qui peut par ailleurs s’accompagner de lésions respiratoires, cardio-vasculaires et neurologiques en rapport avec les lésions associées. La connaissance de ce syndrome, permet de bien prendre en charge ces patients et d’instaurer rapidement un traitement adéquat qui est symptomatique et non spécifique, afin d’éviter l’extension des lésions ischémiques responsables de séquelles neurologiques graves. Le pronostic est généralement bon, il est lié à deux facteurs qui sont la durée de la compression thoracique et la gravité des lésions.

## Conflits d’intérêts

Les auteurs ne déclarent aucun conflit d’intérêts.
